# Redifferentiation of Radioiodine Refractory Differentiated Thyroid Cancer for Reapplication of I-131 Therapy

**DOI:** 10.3389/fendo.2017.00260

**Published:** 2017-10-12

**Authors:** Chae Moon Hong, Byeong-Cheol Ahn

**Affiliations:** ^1^Department of Nuclear Medicine, Kyungpook National University School of Medicine and Hospital, Daegu, South Korea

**Keywords:** redifferentiation, differentiated thyroid cancer, I-131, radioiodine refractory, NIS

## Abstract

Although most differentiated thyroid cancers show excellent prognosis, treating radioiodine refractory differentiated thyroid cancer (RR-DTC) is challenging. Various therapies, including chemotherapy, radiotherapy, and targeted therapy, have been applied for RR-DTC but show limited effectiveness. Redifferentiation followed by radioiodine therapy is a promising alternative therapy for RR-DTC. Retinoic acids, histone deacetylase inhibitors, and peroxisome proliferator-activated receptor-gamma agonists are classically used as redifferentiation agents, and recent targeted molecules are also used for this purpose. Appropriate selection of redifferentiation agents for each patient, using current knowledge about genetic and biological characteristics of thyroid cancer, might increase the efficacy of redifferentiation treatment. In this review, we will discuss the mechanisms of these redifferentiation agents, results of recent clinical trials, and promising preclinical results.

## Introduction

Radioiodine has been used for diagnosing and treating differentiated thyroid cancer (DTC) for more than 70 years. The sodium iodine symporter (NIS) plays a critical role in radioiodine accumulation in DTC cells. The NIS is a membrane glycoprotein that transports two sodium ions and one iodide ion into the cytosol of benign and tumorous thyroid cells from extracellular fluid (1–3). Since radioiodine also can be taken up by the NIS, radioiodine can be used to visualize or selectively kill DTC cells.

Until now, I-131 therapy has been the first-line treatment for unresectable radioiodine-avid metastatic DTC, and radioiodine uptake is a good prognostic marker (4, 5). However, poorly DTC or anaplastic thyroid cancer cells do not express the NIS, and some DTC cells lose expression of the NIS with disease progression (6). Although most patients with thyroid cancer show good prognosis, 1–4% of the patients show distant metastasis at initial diagnosis and 7–23% of the patients show distant metastasis during follow-up periods (7, 8). One-third of metastatic DTC patients do not accumulate radioiodine, and two-thirds of metastatic DTC cases become radioiodine refractory DTC (RR-DTC) (4, 5, 9, 10).

ATA 2015 guideline suggested following criteria as definition of structurally evident RR-DTC: (i) the malignant/metastatic tissue does not concentrate radioiodine; (ii) the tumor tissue loses the ability to concentrate RAI after previous evidence of radioiodine-avid disease; (iii) radioiodine is concentrated in some lesions but not in others; and (iv) metastatic disease progresses despite significant concentration of radioiodine (11). However, there are some differences of detail definitions of RR-DTC according to the researchers, such as number of previous radioiodine therapy, cumulative dose of radioiodine, FDG avidity of the lesion, and so on (12–14). The trivial discrepancy about the definition of RR-DTC originates from generation of the clinical view point, and it can be modified in the future by following clinical experiences.

**Figure 1:**
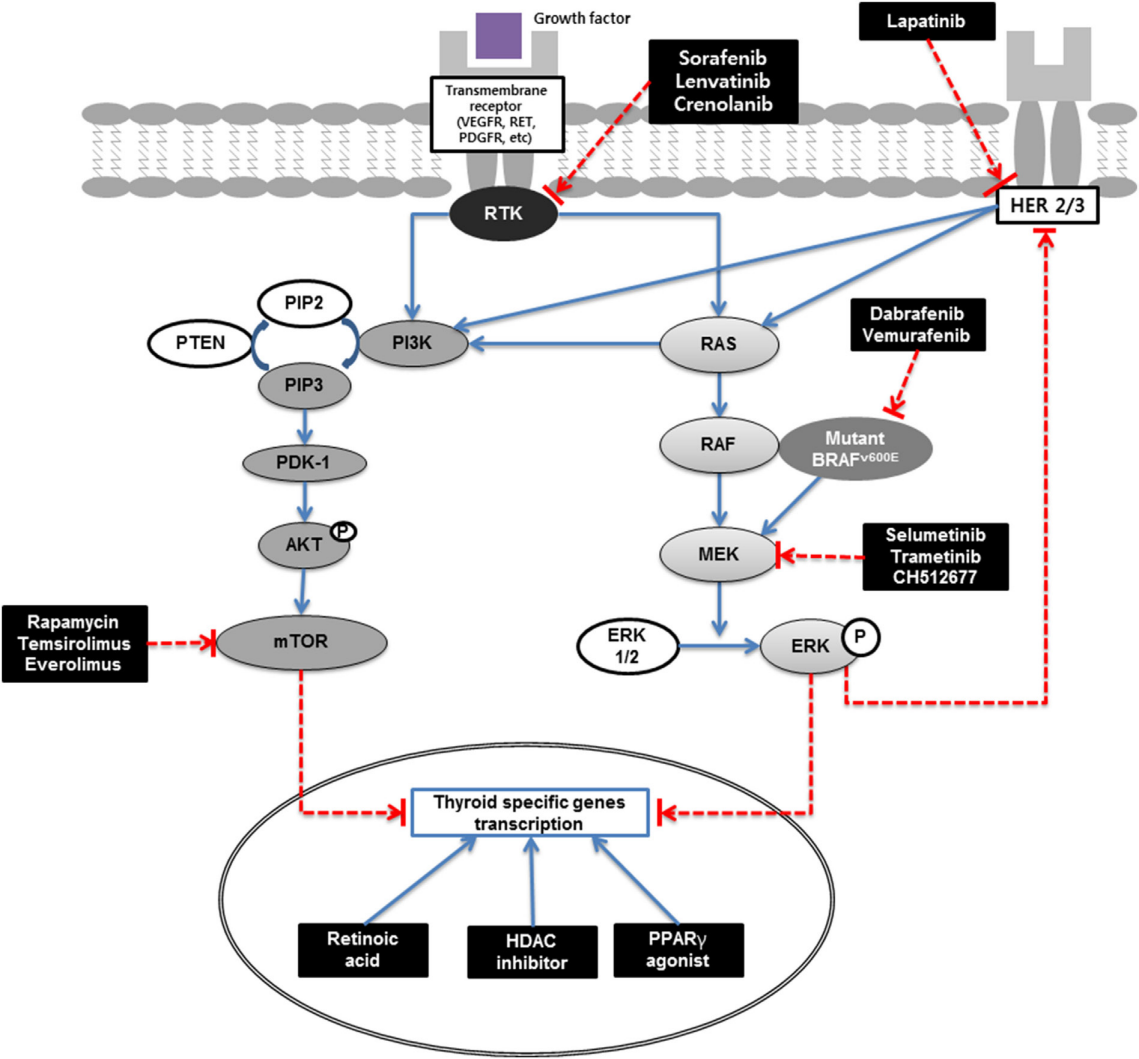
Redifferentiation of thyroid cancer schematic. MAPK (RAS/RAF/MEK) and PI3K/AKT/mTOR pathways are main signaling pathways in thyroid carcinogenesis. Extracellular signals activate RTK and RAS, which in turn activates RAF (mainly BRAF in differentiated thyroid cancer). Activated BRAF phosphorylates and activates the MEK, which in turn phosphorylates and activates ERK. Phosphorylated ERK translocate into the nucleus, where it regulates transcription of the genes involved in cell differentiation, proliferation, survival, and thyroid-specific genes transcriptions. PI3K/AKT activates mTOR which is a key regulator of cell proliferation, inhibitor of apoptosis, and thyroid-specific genes transcriptions. Signaling cascade can be blocked by new targeted therapies. RA binds to nuclear receptors designated as RA receptors (RAR) or retinoid X receptors (RXR). PPARγ agonists bind to RXR and form heterodimers and regulate the transcription of various genes. RAR or RXR complexes bind to the responsive elements in gene promoter sites and activate the transcription of their target genes. HDAC is an enzyme that acetylates histone and silences gene expression; HDAC inhibitors increase gene expression at an epigenetic level. RTK, receptor tyrosine kinase; VEGFR, vascular endothelial growth factor receptor; RET, rearranged during transfection; PDGFR, platelet-derived growth factor receptors; HER, human epidermal growth factor receptor; PI3K, phosphoinositide 3-kinase; PIP2, phosphatidylinositol 4,5-bisphosphate; PIP3, phosphatidylinositol (3,4,5)-trisphosphate; PTEN, phosphatase and tensin homolog; PDK-1, pyruvate dehydrogenase lipoamide kinase isozyme 1; AKT, protein kinase B; mTOR, mechanistic target of rapamycin; RAS, rat sarcoma; RAF, rapidly accelerated fibrosarcoma; MAPK, mitogen-activated protein kinase kinase; ERK, extracellular signal-regulated kinase; HDAC, histone deacetylase; PPARγ, peroxisome proliferator-activated receptor γ.

Radioiodine refractoriness is mainly related to the NIS expression of the thyroid cancer cells. And the ability to concentrate radioiodine is generally considered to indicate a more differentiated phenotype. Inverse relationship between radioiodine and FDG uptake also suggests that the positive correlation between differentiation and radioiodine uptake of the tumor (10). Recent advance of the cancer genetics showed major mutation of papillary thyroid cancer: BRAF V600E accounts for 60%, RAS for 15%, and receptor tyrosine kinase (RTK) for 12% (15). And these RTK and mitogen-activated protein kinase (MAPK) pathway plays a major role in expression of thyroid-specific genes, including NIS (Figure 1). Many other studies also suggested that radioiodine refractoriness is related to MAPK pathway activation (16, 17). Therefore, signaling proteins of the pathway are considered as new targets for redifferentiation.

For RR-DTC, no curative treatment modality is currently available, except complete surgical excision which is not always possible. Usually, RR-DTCs are also refractory to external radiotherapy or chemotherapy (18). Targeted therapies, such as tyrosine kinase inhibitor (TKI) treatment, have emerged as new treatment modalities for RR-DTCs. The Food and Drug Administration of United States and European Medical Agency approved sorafenib and lenvatinib for the treatment of RR-DTCs (13, 14). However, the challenges of using TKI drugs are maintaining the continuous use of these drugs and managing their adverse effects (19). Although many phase III clinical trials on these drugs showed improved progression-free survival and objective response rate, only limited overall survival gains were achieved (13, 14). The escape phenomenon and resistance are other common limitations of TKIs (20).

Redifferentiation therapy followed by I-131 therapy is an alternative option for RR-DTC patients. Previous studies suggested that several compounds such as retinoic acid (RA), MEK/ERK inhibitors, PPARγ agonists, histone deacetylase (HDAC) inhibitors, and phosphatidylinositol 3-kinase (PI3K)/AKT inhibitors have the potential to redifferentiate RR-DTC. Treatment with these compounds resulted in increased radioiodine uptake by enhanced NIS expression in DTC (18), and recent clinical trials showed promising results of redifferentiation therapy followed by I-131 therapy (12, 21). In this review, we will discuss the basic underlying mechanisms of each redifferentiation therapy and results of recent clinical trials and promising preclinical results.

## Retinoic Acid

Retinoic acid is a well-known drug for certain dermatological diseases and has been also used as a redifferentiation agent for hematological malignancies and thyroid cancer for more than 20 years. RA is a metabolite of vitamin A that binds to nuclear receptors designated as RA receptors (RAR) or retinoid X receptors (RXR) (18). RA-RAR or RA-RXR complexes bind to the responsive elements in gene promoter sites and activate the transcription of their target genes. RA is related to cellular differentiation, proliferation, and apoptosis, and induces the redifferentiation and expression of NIS genes in thyroid cancer cells (22). Therefore, radioiodine uptake and thyroglobulin production by thyroid cancers are increased by RA treatment (23).

For dedifferentiated thyroid cancers that show no radioiodine uptake, RA has been used as an alternative treatment. Simon et al. performed the first clinical trial of RA treatment, and observed the renewed uptake of radioiodine in 4 of 10 patients with RR-DTC (24). Other early clinical trials also reported that about 40–50% of patients with RR-DTC showed increased radioiodine uptake (25–27). Fernandez et al. reported that 9/27 (33%) showed optimal radioiodine uptake in metastatic lesions, and 10/27 (37%) showed suboptimal radioiodine uptake after RA treatment. Among them, 4/9 (44%) of optimal uptake group and 3/10 (30%) suboptimal uptake group showed partial response (PR) or stable disease (SD) (28). Oh et al. reported that 19/47 (40.4%) of patients showed a treatment response [1 complete response (CR), 9 PR, 9 SD] (29).

However, response to I-131 therapy after RA pretreatment did not always correlate with increased radioiodine uptake (27). And subsequent studies showed disappointing results, with only 6–20% of patients showing increased radioiodine uptake upon RA pretreatment (23, 30–33), several studies did not find any clinical value of I-131 therapy after RA pretreatment in patients with RR-DTC (31, 33).

As most of the studies were performed using 13-cis-RA, all-trans-RA, and bexarotene (RXR activator) were also applied for redifferentiation and showed good results (18). Zhang et al. used all-trans-RA and reported that radioiodine uptake was increased in 4/11 patients and 7/11 patients showed a response (5 PR, 2 SD) (34). In addition to increasing radioiodine uptake, all-trans RA may suppress cell proliferation by activating pro-apoptotic pathways or directly influencing the cell cycle (35, 36), increased uptake of radioiodine was not necessarily accompanied by reductions of tumor sizes (34). Liu et al. reported that bexarotene (RXR activator) induced radioiodine uptake in 8/11 patients, but no significant therapeutic effectiveness was demonstrated (37).

The controversy about the effect of RA might be associated with the relatively small number of subjects and heterogeneous molecular features of enrolled tumors. Recently, genetic and molecular analyses (such as tests for BRAF, RET/PTC, and RAS, among others) revealed diverse molecular characteristics of thyroid cancers, but most clinical trials applying RA did not analyze these factors. Therefore, more studies are required to unravel the values of RA pretreatment for I-131 therapy in RR-DTC and specific indications for RA-pretreated radioiodine therapy can be developed by these studies.

## HDAC Inhibitor

Histone deacetylase is an enzyme that deacetylates histones, which results in tightly bound DNA, and silences gene expression. HDAC inhibitors increase gene expression at an epigenetic level. In thyroid cancer, HDAC inhibitor treatment is expected to induce redifferentiation by increasing the expression of thyroid-specific genes such as those for thyroid peroxidase and NIS. In addition, HDAC inhibitor treatment also reduces the proliferation rate of thyroid cancer cells and induces apoptosis (38). Previous preclinical studies showed increased radioiodine uptake by pretreatment with various HDAC inhibitors, such as depsipeptide (romidepsin) (39–41), trichostatin A (42), valproic acid (43), sodium butyrate (44), and suberoylanilide hydroxamic acid (SAHA) (45).

However, clinical trials on HDAC inhibitors for RR-DTC patients yielded disappointing results. Kelly et al. used SAHA in various malignancies, and six metastatic thyroid cancer patients were included (46). In the clinical trial, three patients underwent radioiodine scan, and one patient (33%) showed increased radioiodine uptake by SAHA pretreatment. Amiri-Kordestani et al. performed a phase I clinical trial using romidepsin in 11 RR-DTC patients, but follow-up radioiodine scans did not show a meaningful increase in radioiodine uptake (47). Sherman et al. performed a phase II clinical trial to evaluate the effect of romidepsin in 20 patients with RR-DTC, but only 2/20 (10%) patients showed restored radioiodine uptake and no major response was observed (48). Nilubol et al. performed a phase II clinical trial of valproic acid in RR-DTC patients, but valproic acid did not increase radioiodine uptake in tumors (49).

## PPARγ Agonist

Peroxisome proliferator-activated receptor γ (PPARγ) is a nuclear receptor that forms a heterodimer with RXR and regulates the transcription of various genes, such as those involved in adipogenesis, inflammation, cell cycle control, and apoptosis. The thiazolidinediones, also known as glitazones, which are used to treat diabetes mellitus, are PPARγ agonists. Previous studies showed that PPARγ mRNA is downregulated in papillary thyroid cancer cells compared to the normal thyroid tissue, benign nodule, follicular and anaplastic thyroid cancers (50, 51). Various thiazolinediones showed therapeutic effect for DTC, especially PPARγ overexpressing cancers (50–52).

PPARγ agonists also redifferentiate thyroid cancer cells. Frohlich et al. reported that troglitazone increased NIS expression and radioiodine uptake (53). Park et al. also observed upregulation of NIS mRNA after troglitazone treatment in papillary, follicular, and anaplastic thyroid cancer cells (54). Based on these studies, several clinical trials using PPARγ agonists were performed for RR-DTC. Philips et al. performed a clinical trial using rosiglitazone and observed increased thyroglobulin production but only 1/5 (20%) patient showed faint radioiodine uptake (55). Kebebew et al. reported that 5/20 (25%) patients showed positive radioiodine uptake after rosiglitazone treatment, but this radioiodine uptake did not result in a clinically significant response to RR-DTC on long-term follow-up and there was no reduction in tumor size, as revealed by anatomical imaging studies (56).

The results of early clinical trials using PPARγ agonists were not satisfactory, but several studies showed the possibility of better clinical results when specific patient groups were enrolled. Tepmongkol et al. treated 23 patients with RR-DTC with rosiglitazone. Five (71%) of 7 patients with strong PPARγ-positive staining in biopsy, 1/9 (11%) patients with weak staining, and 0/7 (0%) patients with negative staining showed tumoral radioiodine uptake upon treatment (57). On the contrary, another study showed that there was no relationship between PPARγ expression and effectiveness of PPARγ agonist treatment (54, 58). This inconsistency could have arisen because tumor tissue samples might not exactly represent the tumor, as DTC can contain heterogeneous cell populations and genetic alterations.

A chromosomal rearrangement of the PAX8 gene with PPARγ (PAX8–PPARγ) is observed in about 36–50% of follicular thyroid cancer cases (59–61) and 0.8% of papillary thyroid cancer (15). The PAX8–PPARγ rearrangement is related to the loss of the tumor suppressor function of wild-type PPARγ; however, the mechanism is not fully understood yet. Dobson et al. showed promising results that a PPARγ agonist (pioglitazone) was highly therapeutic and prevented metastasis in mice with thyroid-specific expression of PAX8–PPARγ fusion protein (PPFP) and homozygous deletion of phosphatase and tensin homolog (PTEN) (62). Based on these data, a phase II clinical trial of pioglitazone in RR-DTC that contain the PPFP was started (NCT01655719). As pioglitazone was already approved by FDA and has a better toxicity profile than other targeted agents, such as TKIs, positive results of the trial would indicate that pioglitazone may be a good therapeutic option for patients with RR-DTC (63).

## MAPK Pathway Inhibitors

Recent studies using drugs that selectively inhibit the MAPK pathway showed promising results for restoring radioiodine uptake in RR-DTC (12, 16, 21). NIS expression and radioiodine uptake were markedly decreased in response to inducible expression of a BRAF mutation in a mouse model (16). This study also revealed re-expression of NIS and radioiodine uptake after treatment with a MEK inhibitor (16).

Ho et al. published results of a phase II clinical trial using selumetinib, a selective MEK inhibitor, in RR-DTC patients (12). In their study, increased I-124 uptake was observed in 12 of 20 patients with RR-DTC upon pretreatment with selumetinib. They estimated absorbed doses of target lesions using I-124 PET/CT scans, and 8 of these 12 patients achieved appropriate radioiodine uptake to perform a radioiodine therapy (up to 11 GBq). Among them, five patients showed PR to the radioiodine therapy (four with NRAS mutations and one with BRAF mutation). In this study, five patients with NRAS mutations and nine patients with BRAF mutations were included. In patients with a BRAF mutation, 4/9 patients showed increased iodine uptake, but only 1/9 patient showed optimal increase of I-124 uptake and revealed PR after the radioiodine therapy. However, every patient with an NRAS mutation achieved sufficient I-124 uptake to proceed with the radioiodine therapy and 4/5 patients revealed PR by the radioiodine therapy. The effectiveness of selumetinib for recovering radioiodine avidity in RR-DTC was higher in patients with an NRAS mutation than in those with a BRAF mutation.

A recent trial using dabrafenib, a selective BRAF inhibitor, showed restoration of radioiodine uptake in RR-DTC (21). This trial was performed for patients with BRAF-mutant RR-DTC. Six of 10 patients (60%) developed radioiodine uptake after treatment of dabrafenib. This study did not perform a dosimetric analysis, and they applied an empirical fixed-dose treatment (5.5 GBq). Six months later, five of the six treated patients showed a reduced size of the target lesions by CT imaging (two PR, three SD). Although dabrafenib treatment showed better results than selumetinib treatment in patients with BRAF-mutated RR-DTC, dabrafenib still showed relatively poorer response than selumetinib for NRAS-mutated RR-DTC.

The discordant result observed between patients with RAS-mutated and BRAF-mutated DTCs might be associated with the different amount of activation of the MAPK pathway. The higher MAPK activation is related to the lower expression of NIS. BRAF-mutated DTCs showed higher MAPK activation and lower expression of NIS than RAS-mutated DTCs (16, 17). BRAF-mutated cancers and those that were positive for FDG uptake are often refractory to radioiodine therapy (10, 64). BRAF-mutated papillary thyroid cancers showed lower expression of thyroid-specific genes, which are related to iodine accumulation, than RAS-mutated papillary thyroid cancers (15). Since BRAF mutation suppresses the expression of these genes and inhibits radioiodine uptake (Figure 1), RAF or MEK inhibitors can partially restore the response to radioiodine therapy (16, 17). Another explanation for the poor outcomes of BRAF-mutated DTC is the rebound of MAPK signaling after treatment with BRAF or MEK inhibitors (65).

Based on the promising results of preliminary selumetinib treatment (12), the SEL-I-METRY trial (EudraCT no. 2015-002269-47) was initiated to confirm the previous results. This multicenter phase II single-arm study will recruit 60 RR-DTC patients. Pretreatment biopsy and baseline I-123 SPECT/CT will be performed, followed by 4 weeks of selumetinib administration. Then, I-123 SPECT/CT will be performed again to determine the changes of radioiodine accumulation in RR-DTC and to evaluate radioiodine tumor dosimetric analysis. Patients who are considered suitable for radioiodine therapy will receive 5.5 GBq I-131. A further series of post-therapy SPECT/CT scans will be performed to calculate the absorbed dose. The primary endpoint for this trial is progression-free survival at 12 months in RR-DTC patients. Exploratory analyses will include lesional dosimetry; examination of BRAF, RET, and RAS mutation status and protein-bound iodine as potential biomarkers; and quality of life. If SEL-I-METRY demonstrates clinical benefits of this treatment schedule, a phase III trial comparing this strategy with other emerging treatment options will be considered (66).

The appropriate I-131 dose can be determined by empiric and dosimetric approaches, and until now, the superiority of one approach over the other is under debate (67, 68) regarding whether I-123/I-131 SPECT/CT or I-124 PET/CT provides more valuable results to calculate lesion dosimetry (69–71). Ho et al. used I-124 PET/CT for dosimetric analysis (12), but Rothenberg et al. used a fixed-dose approach (21). As I-124 is not available in many hospitals, SPECT/CT could be more commonly used for dosimetric analysis. Studies that compare the effectiveness of empiric and lesional dosimetry to determine the dose of I-131 after redifferentiation treatment are also needed.

Two other randomized, placebo-controlled clinical trials have been initiated in United States. The ASTRA study was designed to determine whether selumetinib followed by I-131 therapy prevents disease recurrence following initial surgery in patients with high-risk DTC (NCT01843062). The other clinical trial aims to determine the response rate at 6 months following treatment with I-131 in combination with placebo or selumetinib for radioactive iodine-avid recurrent and/or metastatic thyroid cancer (NCT02393690). These two studies focus on increasing the effectiveness of I-131 therapy and might dramatically alter future radioiodine therapy paradigms. Moreover, a short duration of TKI treatment before I-131 therapy might reduce costs and toxicity by enhancing therapeutic effectiveness (72).

## Preclinical Promising Targets for Redifferentiation

As previously mentioned, many targets for redifferentiation of RR-DTC have been suggested (Table 1), but no CR has been observed yet. Therefore, many researchers are trying to figure out new targets of redifferentiation in RR-DTC.

**Table 1:** Redifferentiation target mechanism and candidate drugs.

Target mechanism of redifferentiation	Candidate drugs
**Modulating gene transcription**	
RA	13-cis-RA, all-trans-RA, bexarotene (RXR activator)
HDAC inhibitor	Depsipeptide (romidepsin), trichostatin A, sodium butyrate, SAHA, valproic acid, curcumin
PPARγ	Thiazolidinediones (glitazone; troglitazone, rosiglitazone, pioglitazone)
**MAPK pathway**	
RAF	Dabrafenib, vemurafenib
MEK	Selumetinib, trametinib, CH5126766 (CKI)
**Receptor tyrosine kinase**	
PDGFRα	Crenolanib
HER3	Lapatinib
**Autophagy activator**	
PTEN	Antisense-miR-21
mTOR	Rapamycin, temsirolimus, everolimus
Intracellular calcium	Digitalis-like compound, curcumin

*RA, retinoic acid; RXR, retinoid X receptor; HDAC, histone deacetylase; SAHA, suberoylanilide hydroxamic acid; PPARγ, peroxisome proliferator-activated receptor γ; MAPK, mitogen-activated protein kinase; RAF, rapidly accelerated fibrosarcoma; MEK, MAPK kinase; PDGFR, platelet-derived growth factor receptors; HER, human epidermal growth factor receptor; PTEN, phosphatase and tensin homolog; mTOR, mechanistic target of rapamycin*.

Receptor tyrosine kinase is one of the key regulators of redifferentiation. Recently developed targeted agents are targeting this RTK family, including vascular endothelial growth factor receptor, rearranged during transfection (RET), platelet-derived growth factor receptors (PDGFRs), human epidermal growth factor receptor (HER), and so on. Recently developed targeted agents for RTK are applied for redifferentiation of RR-DTCs. Lopez-Campistrous et al. found an inverse relationship between PDGFRα activation and the transcriptional activity of thyroid transcription factor-1 (TTF1). As TTF1 promotes NIS expression, PDGFRα blockade restored NIS expression (73).

Silenced NIS gene in RR-DTC is associated with activation of the MAPK pathway, which is frequently related with the BRAF V600E mutation (17). Therefore, selective BRAF and MEK inhibitors have been applied to redifferentiation and to enhance radioiodine uptake in RR-DTC (12, 74), but rebound MAPK pathway activation through HER3 activation was observed (65). BRAF or MEK inhibitors release transcription repressor proteins from the HER3 promoter and induce HER3 gene expression. HER expression was significantly increased in five of six thyroid cancer cell lines and increased HER3 activates PI3K and MAPK pathways (65). Combination therapy of BRAF/MEK inhibitors (dabrafenib/selumetinib) with a HER inhibitor (lapatinib) resulted in MAPK pathway suppression without a rebound phenomenon and increased NIS expression (75). A phase I study of dabrafenib in combination with lapatinib in BRAF-mutated thyroid cancer had been initiated (NCT01947023). If this study and the following phase II/III studies show promising results, the combined use of BRAF/MEK inhibitors with lapatinib could be a treatment option for patients with RR-DTC.

To overcome the rebound of MAPK signaling, an allosteric MEK inhibitor (CH5126766, known as CKI) could be a new candidate. CKI binds selectively to the non-phosphorylated form of MEK, and stabilizes RAF/MEK complex, which is inactive and stable. Thus, the CKI suppresses the feedback induction of MEK phosphorylation that occurs after ERK pathway inhibition in tumors exposed to other MEK inhibitors (76). Nagarajah et al. reported that CKI treatment restored NIS expression and radioiodine accumulation fivefold and twofold higher, respectively, than selumetinib treatment. Additionally, selumetinib or CKI treatment prior to radioiodine therapy significantly reduced tumor size compared to the effect of radioiodine therapy without pharmacological pretreatment in a mouse model (77).

Autophagy is a degradation machinery for recycling of intracellular content, and it plays important roles in cancer initiation and progression by influencing proliferation, differentiation and anticancer therapy resistance (78). Interestingly, loss of autophagy activity was shown to be associated with dedifferentiation of thyroid cancer and reduced clinical response to radioiodine therapy (79). PI3K/AKT/mechanistic target of rapamycin (mTOR) inhibitors, calcium channel antagonists, cAMP antagonists, inositol monophosphatase inhibitors, and other targets are considered as autophagy inducers (80).

The PI3K/AKT/mTOR pathway, which is related to downstream of RTK and autophagy activation, is a good therapeutic target for redifferentiation. PI3K acts as a negative regulator of NIS expression, and PTEN is an important positive regulator of NIS expression (Figure 1). miR-21 suppresses PTEN and acts as an oncogenic miR that antisense-miR-21 successfully increased NIS expression (81). The mTOR is considered as a key regulator of multiple downstream pathways that act on basic biological processes of protein synthesis, cell division, and cell death (82). Inhibiting mTOR induces radioiodine uptake through increased TTF1 expression (83, 84). Recently, several clinical trials have been performed to evaluate the effect of an mTOR inhibitor (everolimus) and the combined used of a TKI (sorafenib) and an mTOR inhibitor (temsirolimus) (85, 86). These clinical trials appear to be effective, but they did not evaluate the changes of radioiodine uptake and effectiveness of combined radioiodine therapy. Further clinical trials might be needed to elucidate the changes of radioiodine uptake and radioiodine treatment effect after suppression of PI3K/AKT/mTOR pathway.

Tesselaar et al. demonstrated that digitalis-like compounds, also known as autophagy activators, restore NIS expression and iodide uptake in thyroid cancer cell lines (87). Upregulation of NIS was mediated by intracellular Ca^2+^ and FOS gene activation; FOS binds to the NIS upstream enhancer in conjunction with PAX8 (88). Schwertheim et al. showed potential role of curcumin to enhance the radioiodine therapy (89). Curcumin inhibits HDAC activity (90) and induces autophagy activation (91). Curcumin increased expression of thyroid-specific genes, such as NIS and thyroglobulin in thyroid cancer cell lines. And it reduced proliferation and induced apoptosis (89).

Gao et al. showed that inhibiting bromodomain-containing protein 4 (BRD4), which binds to acetylated histones, results in cell cycle arrest and enhanced radioiodine uptake in thyroid cancer cells (92). Singh et al. showed that inverse agonist of estrogen-related receptor-gamma (ERRγ) enhances NIS expression and radioiodine uptake (93). However, BRD4 inhibitor and inverse agonist of ERRγ need further studies to verify the mechanism of redifferentiation and evaluate optimal increase of radioiodine uptake *in vivo*.

## Conclusion

Classic thyroid cancer redifferentiation agents such as RA, HDAC inhibitor, and PPAR gamma agonist have shown disappointing results in recent clinical trials. However, these studies did not perform tumor characterization, and appropriate selection of redifferentiation agents in each patient, using recently advanced knowledge about genetic and biological characteristics of thyroid cancer, might increase the effectiveness of redifferentiation treatment. Recent clinical trials showed promising results of RAF/MEK inhibitors for redifferentiation of RR-DTC; further verification of the results might change the paradigm of RR-DTC treatment. New target agents and combination use of drugs for redifferentiation in appropriately selected patients might enhance efficacy of radioiodine therapy and improve outcome of RR-DTC, which is refractory to conventional radioiodine treatment.

## Review Criteria

PubMed was searched for articles published from 1997 to July 2017 using the search terms “thyroid cancer,” “redifferentiation,” “retinoic acid,” “PPAR,” “HDAC,” “TKI,” “radioiodine,” “I-131.” Only English language, full-text papers were included. References found in these publications provided additional papers to search and cite.

## Author Contributions

Both CH and B-CA participated in drafting, writing, and editing the manuscript.

## Conflict of Interest Statement

The authors declare that the research was conducted in the absence of any commercial or financial relationships that could be construed as a potential conflict of interest.
